# Supplementary material for: The Construction and Exploration of a Comprehensive MicroRNA Centered Regulatory Network in Foxtail Millet (Setaria italica L.)
Source: Front Plant Sci. 2022 May 6;13:848474. doi: 10.3389/fpls.2022.848474 (PMC9121102; doi:10.3389/fpls.2022.848474)
Supplement: Supplementary file 1 [file Data_Sheet_1.zip › Sit-miR169f_Seita.2G038100.1_227_TPlot.pdf]

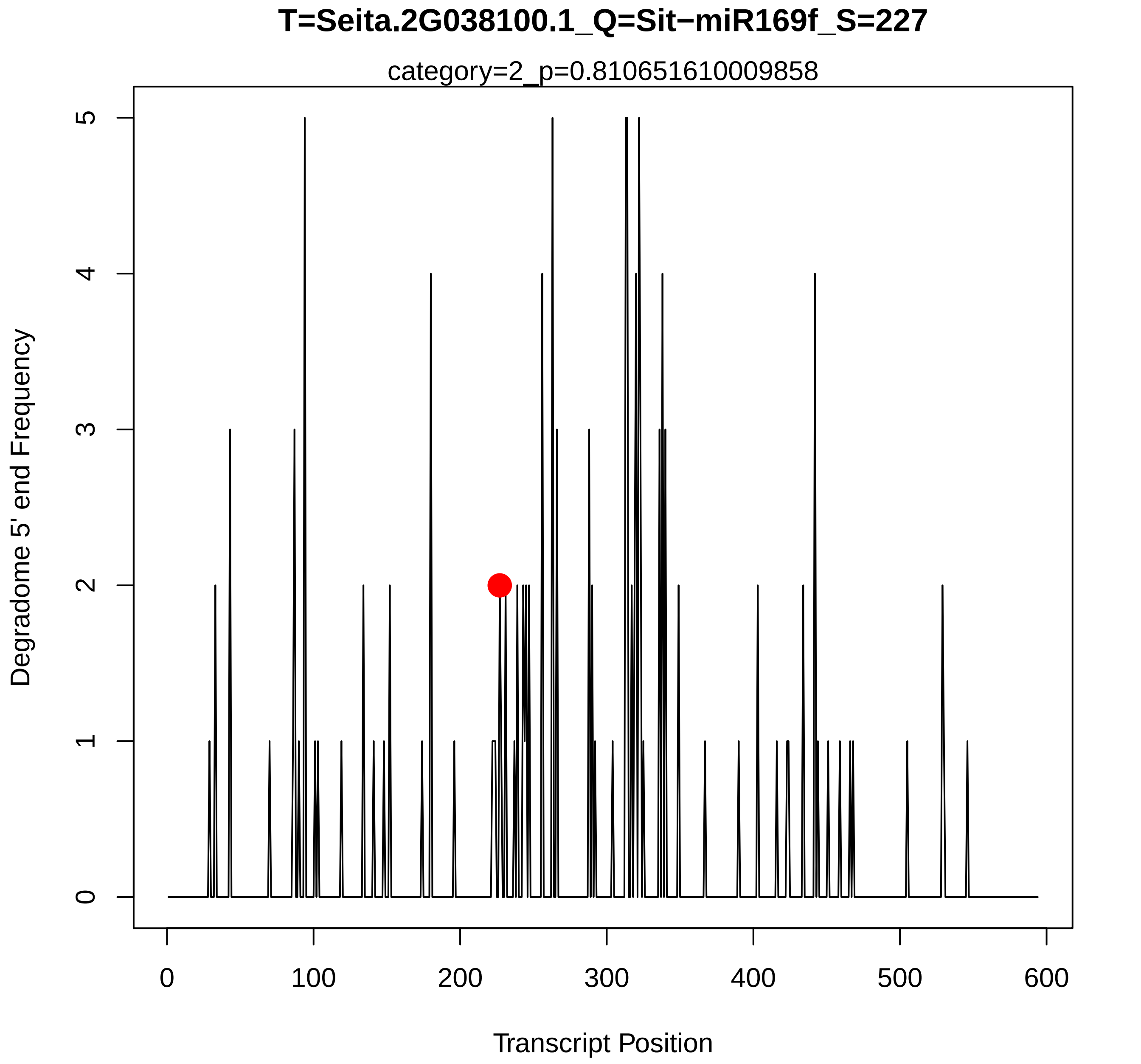

T=Seita.2G038100.1_Q=Sit−miR169f_S=227
category=2_p=0.810651610009858
Degradome 5' end Frequency
0
1
2
3
4
5
0
100
200
300
400
500
600
Transcript Position
